# Optimizing Alternative Substrate for Tomato Production in Arid Zone: Lesson from Growth, Water Relations, Chlorophyll Fluorescence, and Photosynthesis

**DOI:** 10.3390/plants12071457

**Published:** 2023-03-27

**Authors:** Samir Aydi, Sameh Sassi Aydi, Asma Marsit, Nadia El Abed, Rami Rahmani, Jalloul Bouajila, Othmane Merah, Chedly Abdelly

**Affiliations:** 1 Laboratory of Biodiversity and Valorisationof Bioresources in Arid Zones (LR18ES36), Faculty of Sciences, University of Gabes, Gabes 6072, Tunisia; 2 Technical Center of Protected and Geothermal Crops, Avenue AboulkacemChabbiCité El Manara, Gabes 6011, Tunisia; 3 Laboratory of Biodiversity of Actives Biomolecules (LR22ES02), Higher Institute of Applied Biology Medenine, University of Gabes, Medenine 4119, Tunisia; 4 Laboratoire de Génie Chimique, Université de Toulouse, CNRS, INP, UPS, 31062 Toulouse, France; 5 Laboratoire de ChimieAgro-Industrielle (LCA), Université de Toulouse, INRAe, INPT, 31030 Toulouse, France; 6 Département Génie Biologique, IUTA, Université Paul Sabatier, 32000 Auch, France; 7 Laboratory of Extremophile Plants, Center of Biotechnology of BorjCedria, P.O. Box 901, Hammam-Lif 2050, Tunisia

**Keywords:** date-palm compost, soilless cultures, tomato fruit, water relation, photosynthesis, chlorophyll fluorescence, phytochemical analysis

## Abstract

Soilless culture is considered the mostpromising, intensive, and sustainable approach with various advantages for plant production in terms of saving water and nutrients. It can provide consumers with sufficient and high-quality food. However, the commonly used growing substrate for soilless cultivation, coconut fiber (CF), is usually imported and expensive or even unavailable. The objectives of this study were to investigate the impact of local organic farm resources substrates on tomato (*Solanum lycopersicum* L.) plant growth, water relations, photosynthesis, chlorophyll fluorescence, and phytochemical analysis of fruits in a hydroponics culture system. Two growth substrates were evaluated: date-palm waste composted with animal manure (7:3 *w*/*w*) (DPAM) and date-palm trunk compost (DPT). CF and local soil were utilized as positive and negative controls, respectively, in randomized blocks. The results revealed that DPAM substrate enhanced plant growth and physiology: shoot development, leaves tissues hydration, and photosynthetic parameters, as well as chlorophyll fluorescence. However, DPT and CF improved fruit quality: water, mineral, sugar, and protein content. The antioxidant activity of the fruit extract was the greater in DPAM, reaching 13.8 mg GAEg^−1^ DW. This value wasdecreased in soil by 40%. Photosynthesis activity was the most important in DPAM with 12 µmol CO_2_ m^−2^ s^−1^, and only 6.4 µmol CO_2_ m^−2^ s^−1^ in the soil condition. However, regarding the non-photochemical quenching, the dissipated light energy was greater in soil (0.096 ± 0.02) than in DPAM (0.025 ± 0.04). Date-palm waste-based substrates improved tomato vegetative growth and fruit quality as compared to soil-based culture. Date-palm waste-based substrates supplemented with manure appear to be promising and less expensive alternatives to the coconut fiber substrate extensively used in soilless crops in North Africa.

## 1. Introduction

The tomato (*Solanum lycopersicum* L.), which is cultivated on more than 4.8 million hectares of cropland, is a popular vegetable crop around the world [1]. In Mediterranean areas, it is one of the most important field and greenhouse vegetable crops [2]. In order to meet the growing demand for vegetables with higher quality, health benefits, and ecological features, greenhouse tomatoes have progressively converted to soilless cultivation in recent years [3].

Recurring droughts across the worldwide, and especially in the Mediterranean region over the recent decades, have been responsible for significant socioeconomic and ecological consequences [4]. Climate change could exacerbate these problems by increasing the frequency and intensity of extreme weather events such as droughts, heat waves, and cyclones being experienced today [5]. Of great concern are the shifting weather patterns that are impacting food systems. Drought stress is one of the most impactful factors which seriously alter plant physiology, finally leading to a decline in crop productivity [6]. In plants, drought stress causes a set of morpho-anatomical, physiological, and biochemical changes, mainly addressed to limit the loss of water by transpiration through attempting to increase plant water use efficiency [7]. A declined frequency of cell division and cell enlargement, root differentiation, foliage dimensions, shoot length, altered stomatal movements, photosynthetic performance water, and mineral nutrition association with decreased plant yield are major outcomes of drought in plants [8]. In order to overcome these problems and maintain agricultural production, soilless cultivation in greenhouse conditions becomes a necessity from an economic, ecological, and social point of view.

The term soilless culture is defined as the cultivation of plants in systems without soil as a rooting medium, in which the inorganic nutrients absorbed by the roots are supplied via irrigation water [9]. In recent years, a multitude of innovative cultivation processes using bags, mats, and containers, in addition to nutrient solutions, have been developed [10]. These farming methods include systems without a solid substrate, as well as aggregate systems, in which inorganic or organic molecules are used. The benefits of this system include the lack of soil-borne pathogens, a secure substitute for soil disinfection, better use of nutrients and water by plants which reduces wastage, and optimum growing conditions.

Coconut fiber (CF) has been used for decades as a soil substitute in soilless cultivation [11]. In this cultivation system, the yield and quality of horticultural crops can be significantly improved compared to conventional soil culture by managing the quantity and composition of the nutrient solution, as well as the growing medium [12]. Nevertheless, CF substrate is not frequently displayed as it is exported from tropical regions and can spread diseases. For ecological and economic reservations, this had prompted the hunt for substitute materials, particularly organic ones made from local resources. Subsequently, date palm (*Phoenix dactylifera* L.), which belongs to the same family as coconut, has a high resemblance with the fiber of coconut fruit shell [13] and is a widely available product in Tunisia and North Africa. These findings imply that date-palm residues might be used as a suitable alternative for CF in the future.

There are around 105 million palm trees worldwide and an approximate 3,675,000 tons of waste are dumped annually [14], causing environmental hazards [15,16]. The most frequent types of date-palm waste are fronds, branches, stem bark, and leaves. They are obtained by seasonal cutting of palm trees, which is essentially an agricultural technique, and are discarded with no valorization. As a result, from both an economic and environmental aspect, the use of date-palm residues is a potential project [17].

Palm waste has a better water retention capacity than coco peat and can absorb more than eight times its dry weight [18]. Wastes from date-palm trees appear to be an innovative material in the horticulture sector, to be used as growth media or as an organic fertilizer when used as biochar [16]. Although the performance of date-palm waste as a medium for plant development might be exploited in greenhouse plant production, relatively few investigations have used date-palm waste as an alternative for greenhouse soilless substrates.

Given the scarcity of data on the valorization of date-palm waste and the promising opportunities that compost from date-palm waste may present for plant production as a promising, cheaper, and more efficient substrate to be used in the Mediterranean and Gulf regions to replace the imported CF, the current study aimed to investigate the feasibility of using composted date-palm waste as a growth medium in tomato (*Solanum lycopersicum* L.) greenhouse production. The plant growth, water relation, and photosynthetic performance of tomato plants, as well as the quality of fruits, was analyzed with different substrates: compost of date-palm residues and animal manure (DPAM) and compost of date-palm trunk (DPT) compared to CF and local soil.

## 2. Results

This study examined how three different types of growth substrate (DPAM, DPT, and CF) affected tomato plants’ growth, hydration, photosynthesis, and fruit quality as compared to common growing soil. The main goals were to find an appropriate composition for such a substrate with high growth and yield potentials and to improve tomato fruit quality.

### 2.1. Growth Parameters

According to the data in Table 1, DPAM (manure and waste-palm-added substrate), DPT (waste-palm-added substrate), and CF (coco fiber substrate) improved growth parameters in tomato plants as compared to soil. The results showed that all substrates, especially DPAM, increased the yield components, including steam length (m per plant), steam diameter (cm per plant), leaf number per plant, and inflorescence number per plant. The highest values were displayed in the DPAM and DPT substrates, while the lowest values were exhibited by plants growing on soil which was considered as the control growing substrate. CF remained an intermediate substrate regarding plant growth measured values. It should be noted that varying the growing media had different impacts on plant growth components: steam length seems to be the most affected parameter, while the number of inflorescences appears as to be less impacted.

### 2.2. Water Status of Tomato Leaves

**Figure 1:**
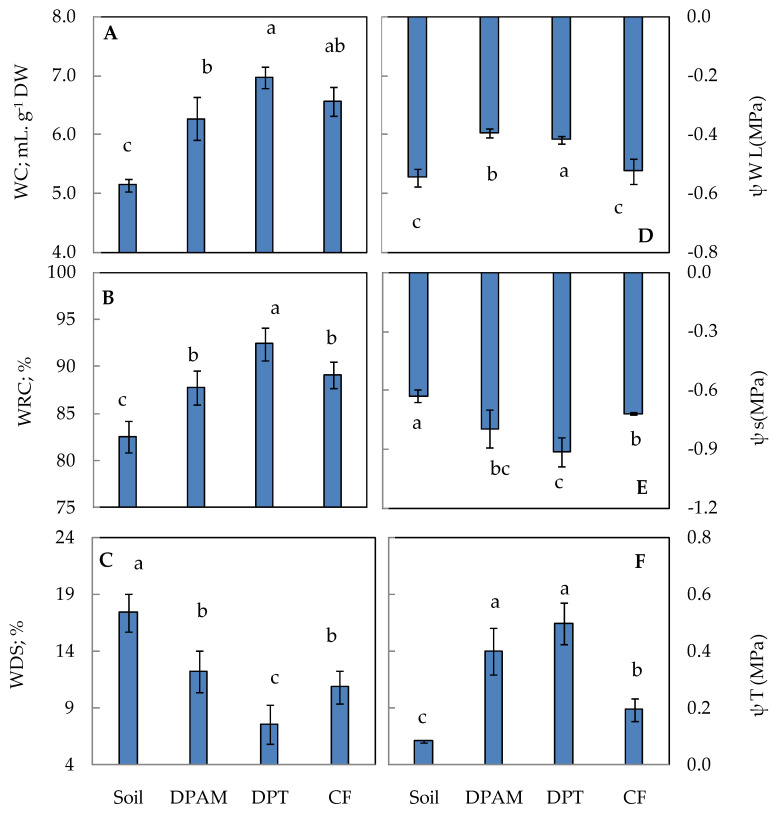
Variation in water parameters in leaves of tomato plants cultivated on different substrates. DPAM: compost of date-palm residues and animal manure; DPT: compost of date-palm trunk; CF: coconut fiber, compared with soil substrate. (**A**): Water content (WC: mL g^−1^ DW); (**B**): relative water content (RWC: %); (**C**): water saturation deficit (WSD: %); (**D**): water potential (Ψw; MPa); (**E**): solute potential (Ψs; MPa); (**F**): pressure (turgor) potential (Ψp; MPa). The plants were 5 months old. The results comprise the mean of six replicates ± S.D. Numbers followed by a different letter within a panel are significantly different at *p* ≤ 0.05, according to LSD analysis.

The water status of leaves was monitored in plants growing in the different studied media (Figure 1). When compared to the soil-growing plants group, tomato plants in the other three groups showed an improved water content (WC in mL gDW^−1^) and relative water content (RWC in %) (Figure 1). This significantly decreased the water saturation deficit (WSD) in the latter plants. Determination of the water potential (ψ_W_), osmotic potential (ψ_S_), and turgor potential (ψ_T_) revealed that, in comparison to soil substrate, the application of palm waste had a stronger impact on preventing water loss in tomato leaves. The coco fiber medium always exhibited an intermediate position.

### 2.3. Leaves Pigment Content Assessment

**Figure 2:**
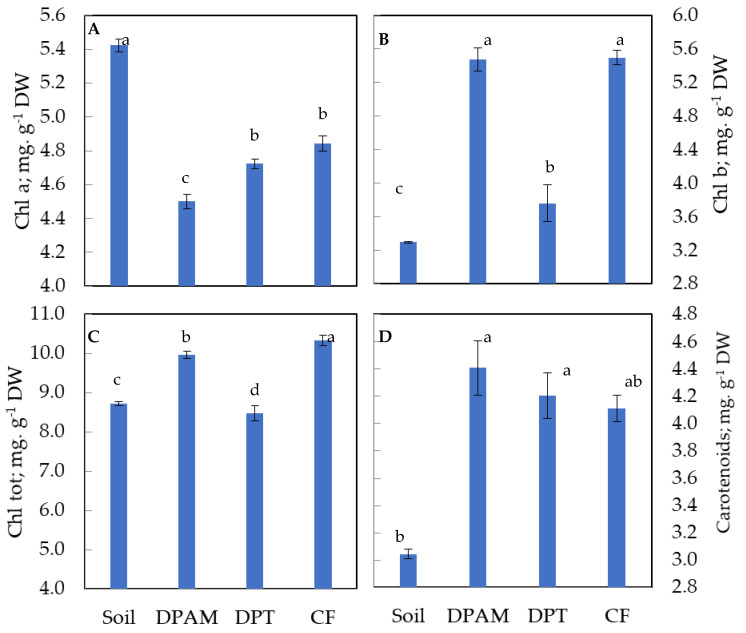
Variation in assimilating pigments content (mg g^−1^ FW) in leaves of 4-month-old tomato plants cultivated on different substrates. DPAM: compost of date-palm residues and animal manure; DPT: compost of date-palm trunk; CF: coconut fiber, compared with soil substrate. (**A**) Chlorophyll-a; (**B**) chlorophyll-b; (**C**) total chlorophyll; (**D**) carotenoids. The results represent the means of six replicates ± SD. Vertical bars indicate standard errors of means. Numbers followed by a different letter within a panel are significantly different at *p* ≤ 0.05, according to LSD analysis.

The data’s statistical analysis (Figure 2) revealed that different substrates had a substantial impact on chlorophyll-a and chlorophyll-b contents. Compared to plants grown in soil, plants grown in other media had considerably lower levels of chlorophyll-a. The DPAM and CF substrates, however, showed higher levels of chlorophyll-b compared to DPT-growing plants, followed by soil-growing plants, which contained the lowest content of chlorophyll-b. Additionally, compared to the control group, the DPAM and CF substrates significantly increased the total chlorophyll content in leaves. DPT and soil exhibited significantly similar contents (8.7 mg g^−1^ DW). The carotenoid content in leaves of different plant groups displayed varying trends: DPAM and DPT displayed the highest values reaching 4.5 mg g^−1^ DW, followed by CF-grown plants. Soil-grown leaves showed the lowest carotenoid contents (3 mg g^−1^ DW).

### 2.4. Photosynthetic Gas Exchange

**Figure 3:**
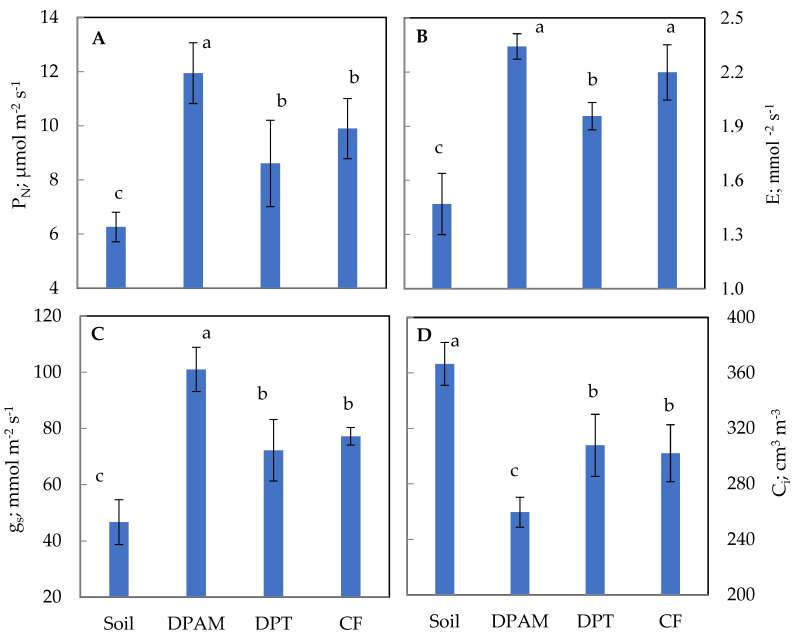
Effects of the culture substrates on photosynthesis parameters in leaves of tomato plants. DPAM: compost of date-palm residues and animal manure; DPT: compost of date-palm trunk; CF: coconut fiber, compared with soil substrate. (**A**): Photosynthetic assimilation rate (P_N_: µmol CO_2_ m^−2^ s^−1^); (**B**): transpiration rate (E: mmol (H_2_O) m^−2^ s^−1^); (**C**): stomatal conductance (gs: mmol (H_2_O) m^−2^ s^−1^); (**D**): intercellular CO_2_ concentration (Ci: µmol (CO_2_) mol^−1^). The plants were 5 months old. The results represent the mean of nine replicates ± S.D. Vertical bars indicate standard errors of means. Numbers followed by a different letter within a panel are significantly different at *p* ≤ 0.05, according to the least significant difference (LSD) analysis.

The photosynthetic assimilation rate (PN) was assessed as µmol CO_2_ m^−2^ s ^−1^ (Figure 3A). DPAM showed the highest level, reaching 12 µmol CO_2_ m^−2^ s^−1^, with soil medium the lowest (6.4 µmol CO_2_ m^−2^ s^−1^), while DPT and CF occupied an intermediate position. Stomatal conductance is a useful key parameter to assess limitations to photosynthesis and growth potential. An identical pattern was also reported for stomatal conductance (gs), attaining 100 mmol H_2_O m^−2^ s^−1^ in DPAM medium (Figure 3C). Concerning internal CO_2_, plants grown on the four media exhibited statically distinct behaviors. Soil-grown plants, DPAM-grown plants, and both DPT- and CF-grown plants showed the highest, lowest, and middle values of intracellular CO_2_, respectively (Figure 3D). The transpiration rate of tomato leaves was impacted by the growing substrate, as illustrated in Figure 3B. Leaf transpiration was more significant in DPAM and CF substrates, reaching almost 2.4 mmol m^2^ s^−1^. With only 1.4 mmolm^2^ s^−1^, this parameter was dramatically reduced with soil (Figure 3B).

### 2.5. Chlorophyll Fluorescence

Five parameters were used to assess how PSII functioned and how its photochemistry changed under different substrates: the maximum quantum yield of PSII (Y), which represents the highest photochemical efficiency or the primary efficiency of light energy transitions; the real quantum yield (φexc), which indicates the transfer efficiency of absorbed photons to the reaction center of PSII; the photochemical quenching coefficient (qP), which indicates the proportion of the opened reaction center; and the yield of electron transport from PSII to PSI (φPSII) and the non-photochemical quenching (NPQ), indicating the dissipated energy in the form of heat (Table 2).Fluorescence increases from the basic state Fo (all reaction centers are open) to a maximum level Fm (all reaction centers are closed) in response to the administration of a pulse of saturating light. Thus, the maximum photochemical quantum yield of PSII (called Y) can be determined. The ɸexc varied significantly amongst the studied media. It was greater in DPT-grown plant leaves (0.663) compared to soil-grown plants leaves (0.618). Both DPT and CF media displayed the same values of ɸexc, about 0.640. The photochemical quenching coefficient (qP) was different in the four studied media: DPAM exhibited the highest value (0.599), while soil showed the lowest (0.488). The quantum yield for electron transport by PSII (ɸPSII) varied with the substrate used for growing tomato plants. It was the highest in palm-waste-added media (DPAM and DPT), while the lowest value was determined in the soil growing medium. The impact of growing substrate on the non-photochemical quenching (NPQ) that dissipated in the form of thermal energy can be observed in Table 2. As compared to the soil growing medium, NPQ in DPAM showed about 75% inhibition. This inhibition did not exceed 62% in the leaves of plants grown on the other studied media.

### 2.6. Physico-Chemical Parameters of Tomato Fruit

It is evident from the findings collected in Table 3 that all tested attributes varied significantly depending on the growing substrates. Fruit water content decreased only in the soil substrate, while it was about equal among the three other substrates. Concerning ash content, the values changed with the growing substrate. It was the highest in the DPT substrate reaching 12.99% and the lowest in soil (8.05%). The lowest fruit pH was recorded in the DPAM substrate, while the highest value was displayed in DPT. The total soluble solids (°Brix) values for tomato fruits from plants cultivated in soil, DPAM, DPT, and CF were 7.12, 8.73, 7.87, and 9.87 °Brix respectively. According to studies on the variations in chemical composition of tomato fruits from plants produced using each medium, the TSS values of fruits harvested from plants grown in the four different growing substrates revealed that fruits were classed in the following descending order: CF > DPAM > DPT > soil. Titratable acidity was the highest in CF and DPT but decreased in soil, and it was the lowest on DPAM. Depending on the growth medium, considerable variations occurred in nitrogen, protein, and sugar contents. Regardless of the manner of fruit cultivation, there were appreciable variations in fruit quality between the substrates. The CF substrate exhibited the highest fruit quality, showing the highest nitrogen, protein, and sugar contents, followed bythe fruits of plants growing on DPT, then on DPAM. The soil substrate displayed the lowest fruit quality regarding protein and sugar contents (Table 3).

**Figure 4:**
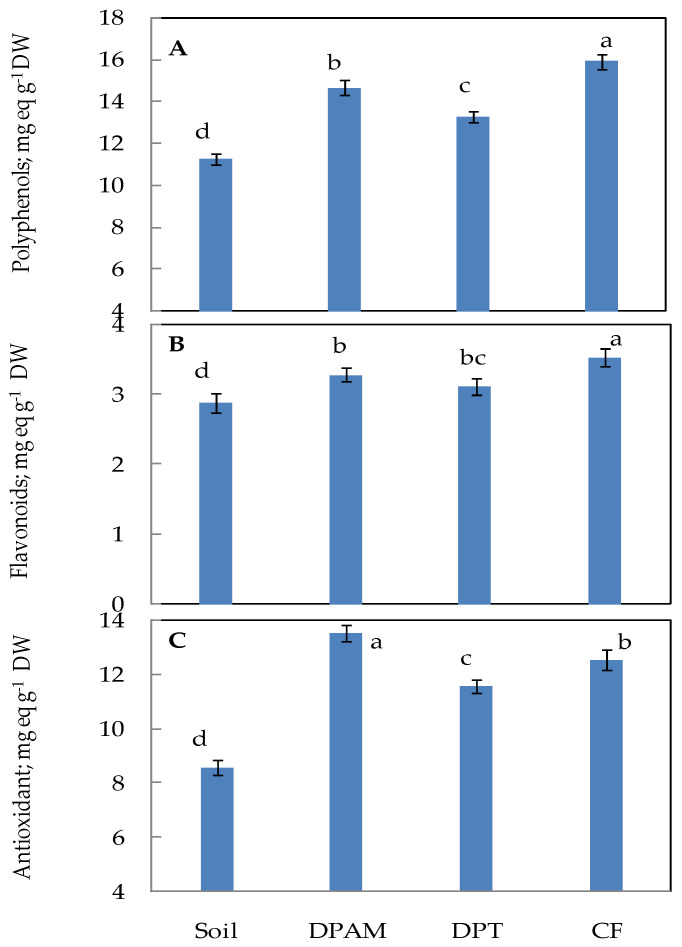
Phytochemical composition and antioxidant activity of extract of tomato fruit grown on different substrates. DPAM: compost of date-palm residues and animal manure; DPT: compost of date-palm trunk; CF: coconut fiber, compared with soil substrate. (**A**): Total polyphenols content; (**B**): total flavonoid content; (**C**): antioxidant activities. Results are expressed as gallic acid or catechin equivalents (mg equivalent g^−1^ DW) and represent the mean of triplicate determinations ± S.D. Vertical bars indicate standard errors of means. Numbers followed by a different letter within a panel are significantly different at *p* ≤ 0.05, according to LSD analysis.

Figure 4 shows that the fruits of plants grown in soil and those harvested from plants cultivated on different growth surfaces have noticeable differences in quality regarding phytochemicals contents and antioxidant activity. The total polyphenols of tomato fruits, as determined by the Folin–Ciocalteu technique, are shown in Figure 4. Polyphenol content was influenced by the growing substrate. According to Figure 4A, fruits grown on the CF substrate had a much greater polyphenol content than fruits from the three other substrates. The soil substrate significantly decreased the total polyphenols content in fruits. The flavonoids content in the samples followed the same pattern as polyphenols (Figure 4B).

Our findings also demonstrated that the tomato extracts had strong antioxidant qualities and that tomato fruits varied in their antioxidant activities (Figure 4C). DPAM displayed the highest activity, reaching 13.8 mg eq g^−1^ DW, followed by CF, and then the DPT substrate—with inhibition reaching 13% and 20%, respectively. Soil seems to decrease this activity in the tested fruits. This inhibition reached 40% as compared to DPAM.

## 3. Discussion

Soilless culture is considered the best system for growing vegetable crops under protected culture in terms of saving water and nutrients. However, the commonly used growing substrate for soilless cultivation, coco-peat, is usually imported and expensive or even unavailable. As a result, there is an increased need to explore cheaper, locally available alternatives to be utilized as growth substrates. To this end, date cultivation is an important sector in North Africa and the Gulf region. Worldwide, an estimated amount of over 3,675,000 tons of residue is discarded annually, leading to environmental problems [14]. Therefore, this experiment compares the growth, physiological parameters, and production of tomatoes grown on date-palm waste and local soil to those grown on a commercial soilless substrate: coco-peat.

The vegetative plant growth parameters of tomatoes after 4 months showed significant variation between the substrates used (Table 1). Organic substrates enhanced the shoot height and stem diameter. DPAM showed the highest value of shoot length by a significant margin as compared to the other media under study. These results corroborate the results of Ravindran et al. [19], who showed a significant increase in the length of PKM I tomatoes under the effect of organic substrates. Stem diameter was most important with the DPAM substrate and less so with the soil media. This was shown by Larounga et al. [20], who reported significant improvements in tomato growth resulting from organic municipal solid-waste compost. The leaf production of tomato plants varied significantly for each of the substrates studied. A significant difference was detected in favor of the date-palm substrates (DPAM and DPT) which presented the highest number of leaves, whereas the least leafy plants were spotted in the soil substrate. Our results are similar to other findings, which have demonstrated the role of compost and manure in significant increases in the number of tomato leaves [21]. The number of flowers during tomato cultivation showed no significant difference between substrates. This demonstrates that the studied substrates had no effect on the number of tomato flowers. This was previously shown by Stoknes et al. [22], who observed no significant effect of organic compost on the tomato flowering yield. However, Darimani et al. [23] found a negative effect of organic waste compost on the number of tomato flowers.

The water content of the leaves provides information on the hydration rate of the tissues. The results of this study showed significant variations in leaf water content depending on the substrate (Figure 1A). These significant differences showed a better hydration of the leaf tissues under the date-palm substrate (DPT) (6.98 mL g^−1^ DW). The lower leaf tissue hydration was spotted in the soil substrate (5.15 mL g^−1^ DW).

The relative water contents of the leaves (RWC, %) reflect the water status of the plant. The RWC values of tomato leaves grown on organic substrates were significantly higher than the control plants (soil) (Figure 1B). Thus, we can deduce that organic substrates have a positive effect on the RWC compared to the soil substrate. Our results are similar to recent studies which have indicated the positive effect of compost on maize and melon water status by increasing foliar RWC [24,25]. Indeed, the high leaf water content reflects the better photosynthesis of the plant, as well as good cellular functioning [26].

The leaf water (LΨw) and osmotic (LΨs) potentials of tomato leaves were significantly influenced by the different growing media. The lowest LΨw was recorded in the soil and CF substrates with −0.545 MPa and −0.524 MPa, respectively (Figure 1D). Indeed, the highest Ψw was detected in the DPT substrate (−0.393 MPa). The LΨs varied among substrates, the lowest in DPAM, then the DPT substrate, and the highest in the soil substrate (Figure 1E). The high LΨw and low LΨs associated with date-palm compost can be explained by the high field capacity and porosity and the water availability for plants from these substrates [25]. Fascella et al. [27] showed that the positive effect of the substrate on plants grown in soilless greenhouse conditions was related to the improvement of water retention. Organic substrates can develop microorganisms promoting plant growth, such as rhizobacteria and mycorrhizae organisms. These benefic microorganism-mediated mechanisms include modifications in the content of plant hormones (e.g., auxin, cytokinin, and gibberellin) and improvement in plant water status by increasing hydraulic conductivity [28,29].

The contents of chlorophyll-a and -b and carotenoids pigments in tomato leaves varied with the culture substrate. The highest total chlorophyll contents, significantly, were detected in the leaves of plants grown on CF (10.34 mg g^−1^ DW) and DPAM (9.97 mg g^−1^ DW). This value was only 8.72 mg g^−1^ DW in soil (Figure 2). The influence of substrate type on total chlorophyll content has previously been studied, showing the positive effect of the date-palm waste substrate on the total chlorophyll content of cucumber and melon compared to the soil substrate [16,25]. Additionally, Ghouili et al. [30] revealed the influence of plant waste compost (date palm) and manure on the increase in total chlorophyll content in tomato leaves. The increase in chlorophyll content helps plants to capture a higher amount of light that supports a higher possibility of *P_N_*due to the conversion of light energy change into chemical energy [31]. Carotenoid pigments accumulated in higher amounts in the leaves of plants grown on organic substrates (Figure 2). This corroborates previous findings, which showed the positive effects of compost or vermicompost on carotenoid content in tomato leaves [19].

The effect of the growing media on the photosynthesis of tomato plants showed significant variations in different photosynthetic parameters (*P_N_*, *gs*, *E,* and *Ci*). The rate of net photosynthesis (*P_N_*) was significantly higher in plants grown on organic substrates than soil. In addition, leaf transpiration rates (*E*) showed a significant increase with organic substrates; the maximum values were observed with DPAM (Figure 3B). Our results are similar to those reported by Jawad et al. [32], which revealed the effect of corn substrate with zeolite on increased *P_N_*, and *E* in tomato. It can therefore be revealed that the results of *P_N_* are positively correlated with the rates of *E*; this relationship can be explained by the stomatal opening, since it is associated with the photosynthetic process by gas exchange [33,34]. Thus, the stomatal conductance (*gs*) was the most important in plants grown on DPAM. Our results are similar to a previous study showing the significant influence of an organic substrate on the increase in *gs* in “Lucius F1” tomato leaves compared to the mineral substrate (rock wool) [35].

The intercellular CO_2_ concentration (*Ci*) is a useful key parameter to assess limitations to photosynthesis potential. *Ci* varies depending on the studied substrate. The lowest concentration was detected in plants grown on DPAM (Figure 3D). This can be explained by the high transpiration flux—where the released water vapor interacts with the CO_2_ molecules and affects their diffusion in the intercellular space—and gives an estimate of the active fixing of CO_2_ by the RuBisCo enzyme in the Calvin cycle [34,36]. 

The fluorescence of chlorophyll was measured in this study. Changes in chlorophyll fluorescence characteristics that may be quantified can reflect the PSII function in response to diverse environmental conditions [37]. The DPAM substrate improved PSII function; the maximal quantum yield of PSII (Y) was much higher when compared to the soil substrate (Table 2), which may be related to plant photosynthetic performance. The decrease in Y on soil can be related to the down-regulation of photosystem II activity and/or impairment of photochemical activity, indicating damage to the photosynthetic apparatus’s functioning [38]. The maximal maximum efficiency of photosystem II (ɸexc) was significantly reduced in soil-grown plants. The photochemical quenching coefficient (qP) results revealed that the PSII reaction centers were partly closed with the soil substrate. Similar findings were observed in drought and salt stress conditions [37,39]. The electron transit from PSII to PSI was altered in soil-cultivated plants, and the light energy was dissipated as heat energy; our non-photochemical quenching (NPQ) data indicated that it was raised by more than 380% when compared to DPAM plants (Table 2).

The DPAM substrate improved the morphological and physiological properties of tomato plants via the amelioration of vegetative growth by increasing leaf size and stem length, as well as photosynthetic activity and water potential. This has previously been shown that a water deficit can affect these parameters [40,41].

In the producing and selling systems, quality is crucial. It is frequently closely related to the conditions of production, the cultivars chosen, plant management, agricultural practices, fruits, photoperiod, etc. These targets can be fulfilled by choosing suitable growth substrates and scheduling fertilization. Numerous variables affect tomato fruit quality, but total soluble solids and pH have the greatest effects, as this study also found. The DPT and CF substrates showed higher fruit hydration, mineral content (ash), titratable acidity, sugar, nitrogen, and protein. These high levels provide information on the good quality of the fruit [42]. Titratable acidity values in this study (0.9–1.2%) were high compared to those reported by Al-Kahtani et al. [43] in the variety “Asala F1” (0.30–0.56%). The positive effect of organic substrate on sugar content has previously been reported in blueberry, strawberry, and melon [25,44,45].

Phytochemical analysis of the fruits showed a positive effect of the organic substrates on the polyphenols and flavonoid contents. This was associated with an increasing antioxidant activity (Figure 4). These results confirm the higher nutritional quality of fruits grown on organic substrates [25,46]. The highest values of antioxidant activity were detected in DPAM fruits. These results agree with other studies on Bemban (*Donax grandis*) and purslane (*Portulaca oleracea* L.) cultivated on organic substrates [47,48].

## 4. Materials and Methods

### 4.1. Experimental Design and Plant Material

This study was carried out in a mono-tunnel greenhouse at the experimental station of the technical center of protected and geothermal crops, Gabès Tunisia.

The experiment was carried out under controlled conditions involving complete randomized blocks with five replicates and four treatments: (1) control (sandy loam soil), (2) compost of date-palm residues added with animal manure (70/30 %) (DPAM), (3) compost of date-palm trunk (DPT), and (4) coconut fiber (CF). Plots were planted with tomato “Murano F1”.

Plants were grown under natural light conditions. They were grown with one stem. The average ambient temperature and relative humidity in the tunnel was in the range of 19.2 ± 1.9 °C and 64 ± 7%, respectively. An open soilless cultivation system was adopted. 

### 4.2. Physicochemical Properties of the Substrates

The chemical conditions of the soil, DPAM, DPT, and CF substrates used were, respectively, in the range of 7.73 ± 0.28, 7.16 ± 0.03, 6.39 ± 0.02, and 7.00 ± 0.11 for the pH; 4.37 ± 0.06, 9.05 ± 0.10, 9.36 ± 0.17, and 8.7 ± 0.38 for the electrical conductivity (EC: dS m^−1^); and 10.38 ± 0.3, 23.1 ± 1.2, 43.1 ± 1.5, and 65.2 ± 4.0 for the field capacity (%). 

Plants were irrigated by the open soilless system irrigation technique. The amount of nutrient solution applied was determined based on a measured drainage fraction. The range of drainage fraction measured was from 20 to 40% during the experimental period. Tomato plants were supplied with the liquid nutrient solution [25].

### 4.3. Plant Growth

Growth parameters were measured on 5-month-old plants. The length of shoots was measured from the base to the top of the plant. Leaves were numbered per plant, and the diameters of each stem was determined at the collar using a vernier caliper.

### 4.4. Leaves Water Parameters

The water content (WC), providing information about the plant tissues’ hydration, was determined by the difference between the fresh weight (FW) and the dry weight (DW).

WC (mL H_2_O. g^−1^ DW) = (FW − DW)/DW

The relative water content (RWC), indicating the actual water content of the leaf tissue as a % of its maximum turgescent capacity, was determined by the difference between the weights of the fresh weight (FW), saturation weight (SW), and the dry weight (DW).

RWC (%) = [(FW − DW)/(SW−DW)∗100]

Leaf water potential (LΨw: MPa) was measured with a Scholander pressure chamber (Model 1505D, PMS Instruments Co., Albany, NY, USA).

The osmotic potential of leaf tissues (LΨs: MPa) was determined by a vapor pressure osmometer of the Wescor type (5500; Wescor Inc., Logan, UT, USA). 

Turgor pressure (Ψp)was estimated as the difference between water potential and osmotic potential.

Ψp (MPa) = Ψw − Ψs

### 4.5. Photosynthetic Parameters:

#### 4.5.1. Extraction, Separation, and Quantification of Pigments

Photosynthetic pigments were extracted with 80% acetone (CH3COCH3). The absorbance of the extracts was measured at 460, 645, and 663 nm using a T80 UV–Vis spectrophotometer (PG Instruments, Leicestershire, UK). The concentrations of chlorophyll-a, chlorophyll-b, and carotenoids pigments (in mg g^−1^ FW) were calculated according to Najar et al. [37].

#### 4.5.2. Gas Exchanges

Leaf gas exchanges were measured on fully developed leaves using a portable CI-340 hand-held photosynthesis system (CI340 Bio-Science, Inc., Camas, WA, USA). The net photosynthesis rate (PN; μmol (CO_2_) m^−2^ s^−1^); stomatal conductance (gs; moles (H_2_O) m^−2^ s^−1^); transpiration rate (E; mmol (H_2_O) m^−2^ s^−1^); and intercellular CO_2_ concentration (Ci; ppm) were determined. The measurements were made at an atmospheric pressure of 103 KPa, an atmospheric CO_2_ level of 550–570 ppm, a relative humidity of 50–55%, a light intensity of 980–1050 mol PAR m^−2^ s^−1^, and a leaf temperature of 21 ± 3 °C.

#### 4.5.3. Chlorophyll Fluorescence

Chlorophyll fluorescence quantifications were performed using a hand-held chlorophyll fluorometer (OS30p+, Opti-Sciences. Inc., Hudson, NH, USA), on leaves previously adapted to the darkness (30 to 40 min). The fundamental state Fo (all reaction centers are open) and the maximum level of fluorescence Fm (all reaction centers are closed) were determined. Then, the maximum photochemical quantum yield of PSII (Y = Fv/Fm = (Fm − Fo)/Fm) was determined. The efficiency of quantum open centers (Φexc = Fv’/Fm′ = (Fm′ − Fo′)/Fm′) was measured just after the transfer of plants into continuous light. In order to estimate the proportion of reaction centers open in the PSII, the coefficient of the photochemical quenching (qP = (Fm′ − Fs)/(Fm′ − F0′)) was allowed. The non-photochemical quenching NPQ ((Fm−Fm’)/Fm’) estimates the dissipated energy in heat form. The quantum yield of electron transport of PSII (ɸPSII = (Fm′ − Fs)/Fm′) estimates the efficiency of all the reaction centers of PSII under light. 

### 4.6. Physicochemical Analyses of Fruits

The moisture (mL g^−1^ DW) was determined by the difference between the fresh weight (FW) and the dry weight (DW) of fruit tissues.

M (% H_2_O) = 100 ∗ [(FW − DW)/FW]

The ash content was estimated after the incineration of fruits at 500 °C for about 3 h in a muffle furnace. 

The total soluble solids (TSS: °Brix) were measured with a digital hand-held refractometer (Model: PAL-1, ATAGO Co., Ltd., Tokyo, Japan).

The total soluble sugars were extracted in 80% ethanol (v v^−1^). The quantification was investigated colorimetrically using a T80 UV–Vis spectrophotometer at 490 nm, according to a modified phenol–sulfuric acid method [49].

The titratable acidity (TA), expressed as a percentage of citric acid, was determined by the titration method as described by Nielsen [50]. 

### 4.7. Preparation of Methanol Extracts 

Dried fruit biomass was extracted in the dark at room temperature by methanol 1:10 (*w*/*v*) for 2 h. After centrifugation (5000 rpm) for 10 min at 20 °C using a HERMLE Z 513 K centrifuge (Hermle Labortechnik, Wehingen, Germany), samples were evaporated using a rotary vacuum evaporator; Büchi Rotavapor R-200 (Büchi Labortechnik, Flawil, Switzerland). The supernatant samples were dissolved in methanol (10 mg mL^−1^).

### 4.8. Total Polyphenols (TPC)and Flavonoids Content (TFC)

TPC was determined spectrophotometrically using the Folin¬–Ciocalteu method [51]. Results were expressed as mg gallic acid equivalents (GAE) g^−1^ of the fruit DW.

TFC was conducted using a colorimetric method described by Sassi Aydi et al. [52]. Results were reported as mg CE/g DW against the calibration curve of catechin.

### 4.9. Total Antioxidant Activity (TAC)

The TAC was determined using the phosphate molybdate method [53]. Results were expressed as mg GAE/g DW.

### 4.10. Statistical Analysis

Data were expressed as the mean value ± SD of 6 replicate samples (3 for phytochemical composition and antioxidant activity). The statistical analyses were performed using the one-way analysis of variance (ANOVA) procedure with the Statistical Package for the Social Sciences (SPSS) version 20.0 software (IBM Corp, released 2011, Armonk, NY, USA). When *p* ≤ 0.05, differences were considered as statistically significant according to Fisher’s LSD test.

## 5. Conclusions

The application of solid-waste compost in agriculture is gaining increased interest, as this can represent not only an effective method of waste management and recycling but also an adequate substrate for soilless culture. In the present study, diverse substrates based on date-palm compost were tested during tomato culture. The use of diverse substrates influenced tomato plant growth, particularly date-palm trunk compost amended with manure (DPAM) which offered the greatest values for stem lengths, diameters, and leaf number. Plant physiological parameters improved when grown on organic substrates rather than soil. The most significant tissue hydration, pigment synthesis, and increased photosynthetic activities were promoted by a local substrate from date-palm trunk compost (DPAM), with the lowest fluorescence and light dissipation.

Concerning the chemical, phytochemical, and organoleptic parameters of the fruits, the use of CF and DPAM substrates allows for better quality in terms of TSS, sugar, pH, protein, polyphenols, flavonoids, and in vitro antioxidant activity. Based on these findings, we can conclude that local DPAM compost can be employed as a suitable substrate for tomato production and fruit quality, in addition to its economic and ecological advantages.

## Figures and Tables

**Table 1:** Growth parameters of three-month-old tomato plants cultivated on different substrates. DPAM: compost of date-palm residues and animal manure; DPT: compost of date-palm trunk; CF: coconut fiber, compared with soil substrate. Numbers followed by a different letter within a line are significantly different at *p* ≤ 0.05, according to the least significant difference (LSD) analysis.

Substrate	Soil	DPAM	DPT	CF
Shoot height (m)	1.50 ± 0.03 c	1.86 ± 0.06 a	1.62 ± 0.07 b	1.71 ± 0.04 b
Stem diameter (cm)	11.60 ± 0.46 b	14.07 ± 0.44 a	13.47 ± 0.50 a	13.80 ± 0.40 a
Leaves number (plant^−1^)	26.20 ± 0.62 bc	27.80 ± 0.82 ab	28.73 ± 0.15 a	27.20 ± 0.86 b
Inflorescence number (plant^−1^)	7.07 ± 0.25 b	7.80 ± 0.40 a	7.13 ± 0.34 ab	7.20 ± 0.40 a

Values are given as the mean ± SD (n = 6); SD: standard deviation.

**Table 2:** Variation in the fluorescence parameters of tomato leaves grown on different substrates. DPAM: compost of date-palm residues and animal manure; DPT: compost of date-palm trunk; CF: coconut fiber, compared with soil substrate. The initial fluorescence (*Fo*), maximum fluorescence (Fm), maximum quantum yield of photosystem II (Y = Fv/Fm), photosystem II maximum efficiency (ɸexc = Fv’/Fm’), photochemical quenching coefficient (qP = Fv’/Fs), quantum yield for electron transport of PSII (φPSII = (Fm’ − Fs)/Fm’), non-photochemical quenching (NPQ = (Fm − Fm’)/(Fm’)).

Substrate	Soil	DPAM	DPT	CF
*F_0_*	133.3 ± 4.33 c	142.6 ± 4.37 ab	155.0 ± 4.47 a	164.3 ± 9.57 a
*F_m_*	571.0 ± 6.91 b	657.3 ± 16.92 a	635.8 ± 23.75 a	626.0 ± 13.57 a
Y	0.767 ± 0.01 b	0.783 ± 0.01 a	0.756 ± 0.01 c	0.737 ± 0.02 d
ɸexc	0.618 ± 0.02 c	0.640 ± 0.02 b	0.663 ± 0.02 a	0.642 ± 0.01 b
qP	0.488 ± 0.02 d	0.599 ± 0.01 a	0.581 ± 0.04 b	0.536 ± 0.07 c
ɸPSII	0.302 ± 0.01 c	0.382 ± 0.07 a	0.387 ± 0.03 a	0.331 ± 0.04 b
NPQ	0.096 ± 0.02 a	0.025 ± 0.04 c	0.036 ± 0.06 b	0.032 ± 0.01 b

Results representthe means of eight replications ± S.D. Numbers followed by a different letter within a line are significantly different at *p* ≤ 0.05, according to LSD analysis.

**Table 3:** Physico-chemical parameters of tomato fruit. Plants were grown on different substrates. DPAM: compost of date-palm residues and animal manure; DPT: compost of date-palm trunk; CF: coconut fiber, compared with soil substrate. TSS: Total soluble solids.

Substrate	Soil	DPAM	DPT	CF
WC (mL g^−1^ DW)	4.71 ± 0.08 b	5.19 ± 0.11 a	5.34 ± 0.09 a	5.28 ± 0.08 a
Ash %	8.05 ± 0.08 d	8.92 ± 0.10 c	12.99 ± 0.07 a	9.18 ± 0.11 b
pH	4.34 ± 0.05 b	4.25 ± 0.05 bc	4.48 ± 0.08 a	4.31 ± 0.05 b
Brix °	7.12 ± 0.10 d	8.73 ± 0.16 b	7.87 ± 0.12 c	9.87 ± 0.16 a
TSS	61.17 ± 1.60 d	71.00 ± 2.10 b	65.33 ± 3.13 c	81.00 ± 3.35 a
Titratable acidity	1.05 ± 0.02 b	0.90 ± 0.02 c	1.16 ± 0.03 a	1.13 ± 0.06 a
Sugar (mg g^−1^ DW)	30.25 ± 1.06 b	31.52 ± 1.20 b	36.88 ± 1.07 a	38.57 ± 1.23 a
Protein (mg g^−1^ DW)	33.54 ± 1.26 d	51.04 ± 0.59 c	53.56 ± 1.15 b	60 ± 1.35 a

Results representthe mean of triplicate determinations ± S.D. Numbers followed by a different letter within a line are significantly different at *p* ≤ 0.05, according to LSD analysis.

## Data Availability

The data will be available on request.
